# Supplementary material for: An Immunological Polysaccharide from Tremella fuciformis: Essential Role of Acetylation in Immunomodulation
Source: Int J Mol Sci. 2022 Sep 8;23(18):10392. doi: 10.3390/ijms231810392 (PMC9499394; doi:10.3390/ijms231810392)
Supplement: Supplementary file 1 [file ijms-23-10392-s001.zip › ijms-1872084-supplementary.pdf]

Supplementary Materials

An Immunological Polysaccharide from *Tremella fuciformis*: Essential Role of Acetylation in Immunomodulation

Tzu-Yin Huang [1], Feng-Ling Yang [1], Hsiao-Wen Chiu [2], Hong-Chu Chao [1], Yen-Ju Yang [1], Jyh-Horng Sheu [3], Kuo-Feng Hua [2] and Shih-Hsiung Wu [1,*]

[1] Institute of Biological Chemistry, Academia Sinica, Taipei 115, Taiwan
[2] Department of Biotechnology and Animal Science, National Ilan University, Ilan 260, Taiwan
[3] Department of Marine Biotechnology and Resources, National Sun Yat-sen University, Kaohsiung 804, Taiwan
* Correspondence: shwu@gate.sinica.edu.tw; Tel.: +886-(02)-2785-5696; Fax: +886-(02)-2653-9142

Table of Contents

Figure S1. The monosaccharide composition analysis of (a) L-fucose, (b) D-xylose, (c) D-mannose, (d) D-glucose, (e) TFP-F1, (f) TFP-F1R, and (g) TFP-F1P. 3
Table S1. GC-MS linkage analysis of the PMAA from TFP-F1 sample 4
Table S2. GC-MS linkage analysis of the PMAA from TFP-F1R sample 4
Table S3. GC-MS linkage analysis of the PMAA from TFP-F1P sample 4
Figure S2. FT-IR spectrum of TFP-F1 5
Figure S3. ^{1}H NMR spectrum of (a) TFP-F1 and (b) deOAc TFP-F1R 6
Figure S4. Full ^{1}H NMR spectrum (800 MHz) of TFP-F1 (D_2O, 323K) 7
Figure S5a. Full ^{13}C NMR spectrum (160 MHz) of TFP-F1 (D_2O, 323K) 8
Figure S5b. Enlarged ^{13}C NMR spectrum (from 55 to 105 ppm) (160 MHz) of TFP-F1 9
Figure S6a. Full HSQC spectrum of TFP-F1 10
Figure S6b. Enlarged HSQC spectrum (I) of TFP-F1 11
Figure S6c. Enlarged HSQC spectrum (II) of TFP-F1 12
Figure S7a. Full HMBC spectrum of TFP-F1 13
Figure S7b. Enlarged HMBC spectrum (I) of TFP-F1 14
Figure S7c. Enlarged HMBC spectrum (II) of TFP-F1 15
Figure S7d. Enlarged HMBC spectrum (III) of TFP-F1 16
Figure S7e. Enlarged HMBC spectrum (IV) of TFP-F1 17
Figure S8. Full COSY spectrum of TFP-F1 18
Figure S9a. Full TOCSY spectrum of TFP-F1 19
Figure S9b. Enlarged TOCSY spectrum of TFP-F1 20
Figure S10a. Full NOESY spectrum of TFP-F1 21
Figure S10b. Enlarged NOESY (I) spectrum of TFP-F1 22
Figure S10c. Enlarged NOESY (II) spectrum of TFP-F1 23
Figure S11. COSY and selective HMBC correlations of TFP-F1 24
Table S4. Key NOE correlations of TFP-F1 24
Figure S12. Full ^{1}H NMR spectrum (800 MHz) of TFP-F1R (D_2O, 298K) 25
Figure S13. Full ^{13}C NMR spectrum (160 MHz) of TFP-F1R (D_2O, 298K) 26
Figure S14. Full ^{1}H NMR spectrum (800 MHz) of deOAc TFP-F1R (D_2O, 298K) 27
Figure S15. Full ^{13}C NMR spectrum (160 MHz) of deOAc TFP-F1R (D_2O, 298K) 28
Figure S16. The standard curve of LAL assay 29
Table S5. Key NOE correlations of TFP-F1 29
Figure S17. The endotoxin quantity of TFP-F1 and its derivatives 30

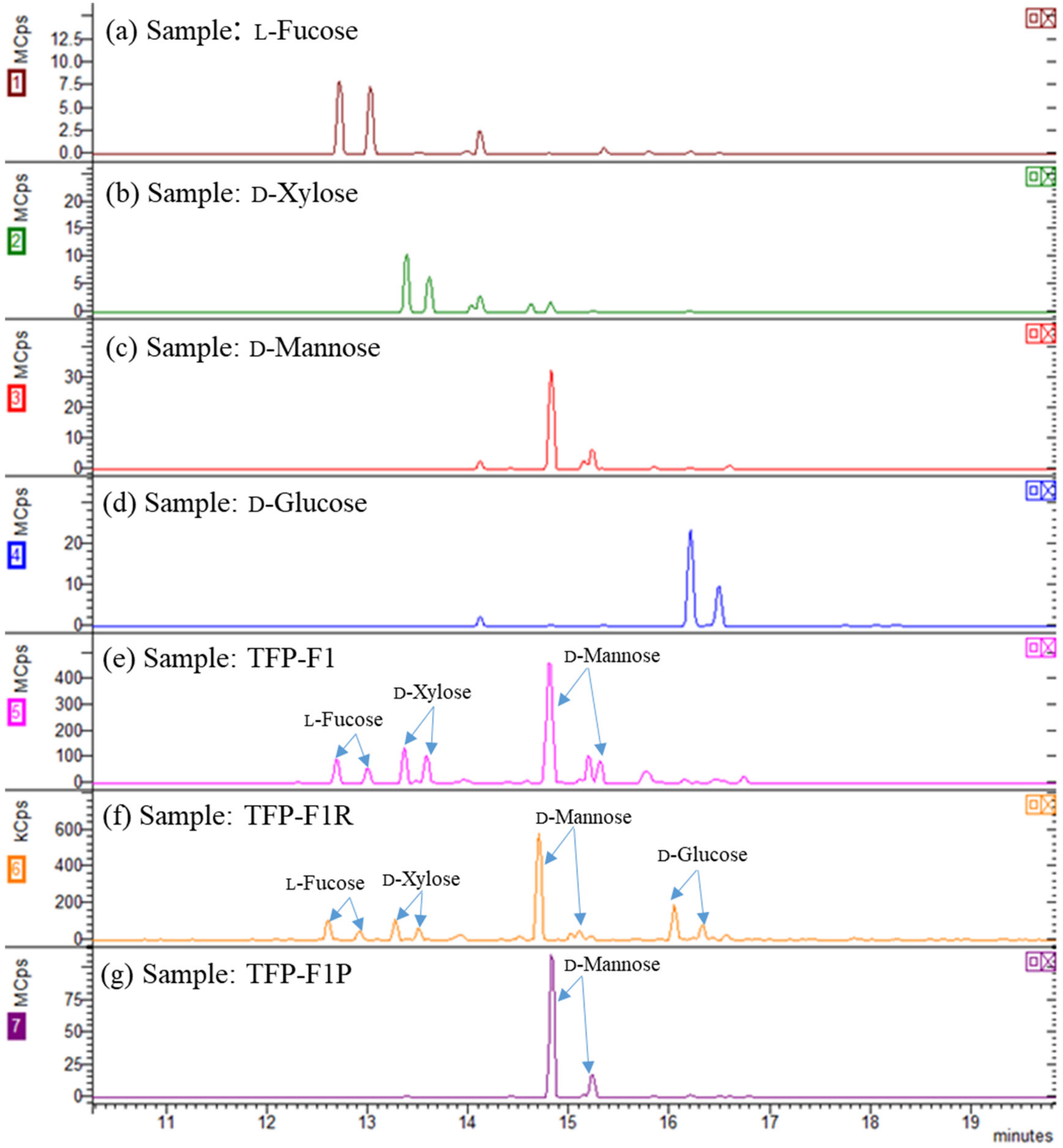

Figure S1. The monosaccharide composition analysis of (a) L-fucose, (b) D-xylose, (c) D-mannose, (d) D-glucose, (e) TFP-F1, (f) TFP-F1R, and (g) TFP-F1P.

Table S1. GC-MS linkage analysis of the PMAA from the TFP-F1 sample

Retention time	Alditol derivative	*m/z*	Linkage type
6.77	6-deoxy-2,3,4-tri-*O*-methyl-L-galactitol	89, 102, 115, 118, 131, 175	Terminal fucose
6.90	3,4-di-*O*-methyl-D-xylitol	88, 101, 117, 130, 190	1,2-linked xylose
7.19	2,4,6-tri-*O*-methyl-D-mannitol	71, 87, 101, 118, 129, 161, 202, 234	1,3-linked-mannose
7.33	4,6-di-*O*-methyl-D-mannitol	71, 87, 101, 129, 161, 202, 262	1,2,3-linked- mannose

Table S2. GC-MS linkage analysis of the PMAA from TFP-F1R sample

Retention time	Alditol derivative	*m/z*	Linkage type
6.77	6-deoxy-2,3,4-tri-*O*-methyl-L-galactitol	89, 102, 115, 118, 131, 175	Terminal fucose
6.90	3,4-di-*O*-methyl-D-xylitol	88, 101, 117, 130, 190	1,2-linked xylose
7.00	2,3,4,6-tetra-*O*-methyl-D-glucitol	71, 87, 102, 118, 129, 145, 161, 162, 205	Terminal glucose
7.19	2,4,6-tri-*O*-methyl-D-mannitol	71, 87, 101, 118, 129, 161, 202, 234	1,3-linked-mannose
7.33	4,6-di-*O*-methyl-D-mannitol	71, 87, 101, 129, 161, 202, 262	1,2,3-linked- mannose

Table S3. GC-MS linkage analysis of the PMAA from TFP-F1P sample

Retention time	Alditol derivative	*m/z*	Linkage type
7.19	2,4,6-tri-*O*-methyl-D-mannitol	71, 87, 101, 118, 129, 161, 202, 234	1,3-linked-mannose

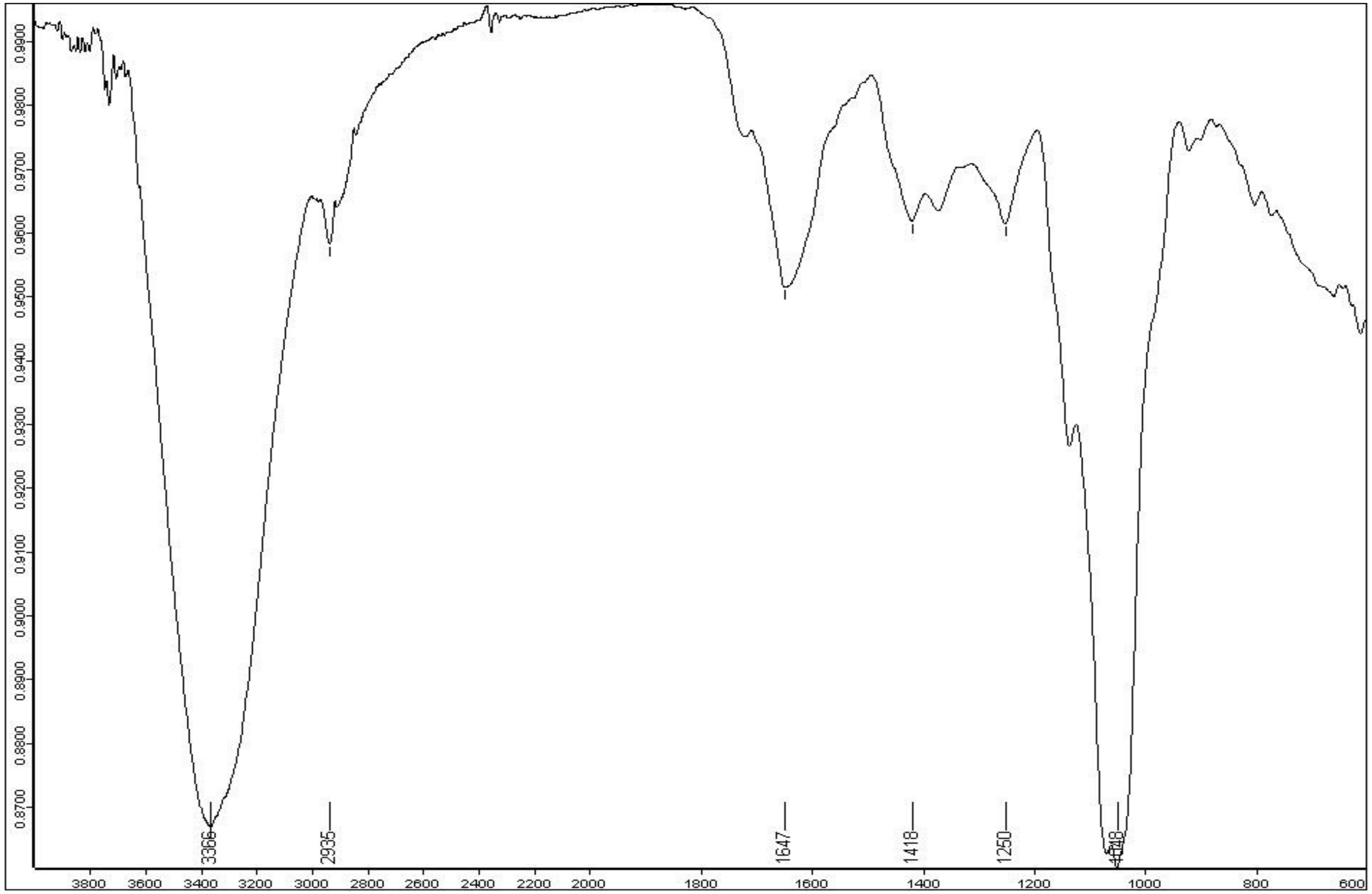

Figure S2. FT-IR spectrum of TFP-F1.

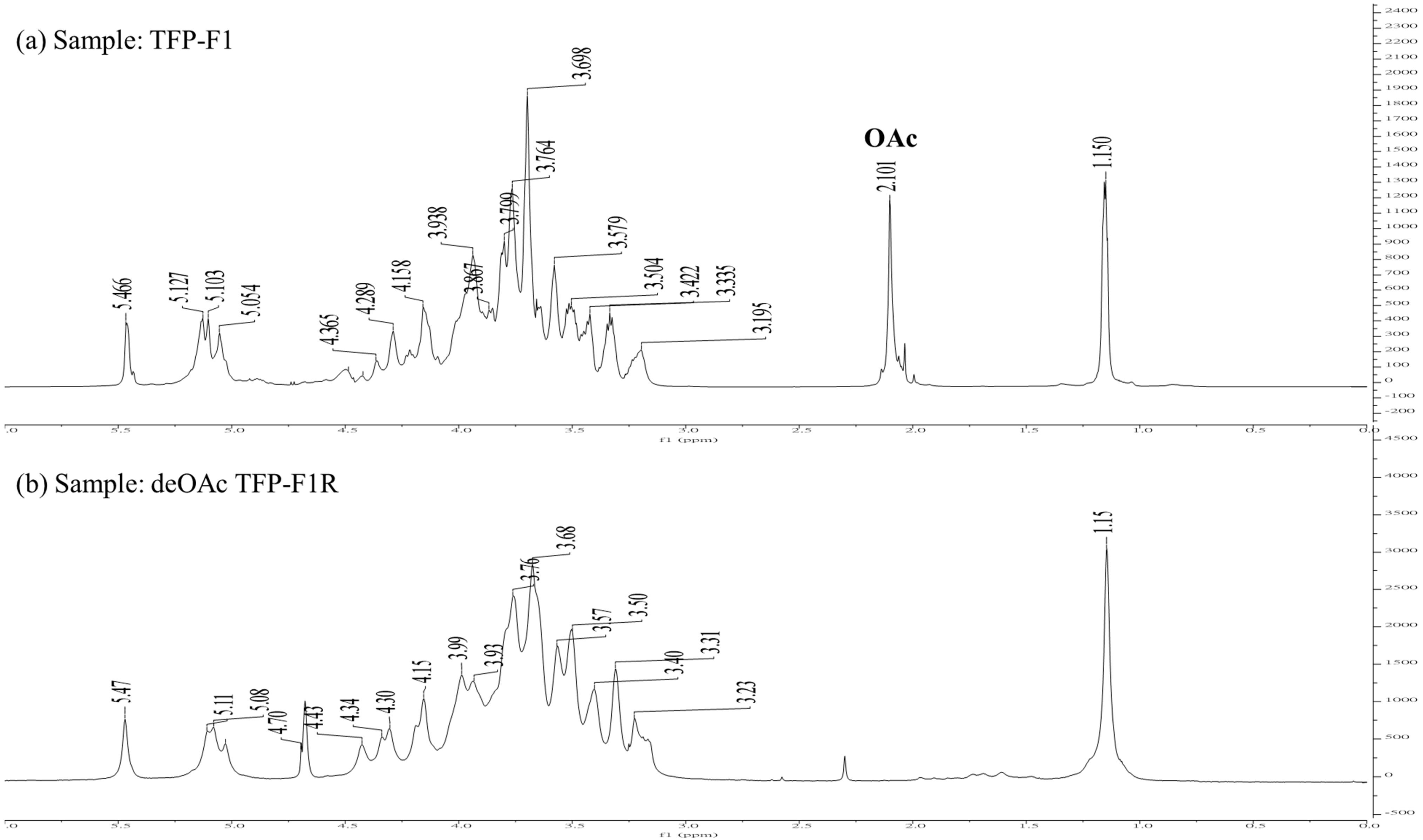

Figure S3. ^{1}H NMR spectrum of (a) TFP-F1 and (b) deOAc TFP-F1R. The sample of deOAc TFP-F1R was prepared by the carboxyl reduction and de-acetylation of TFP-F1.

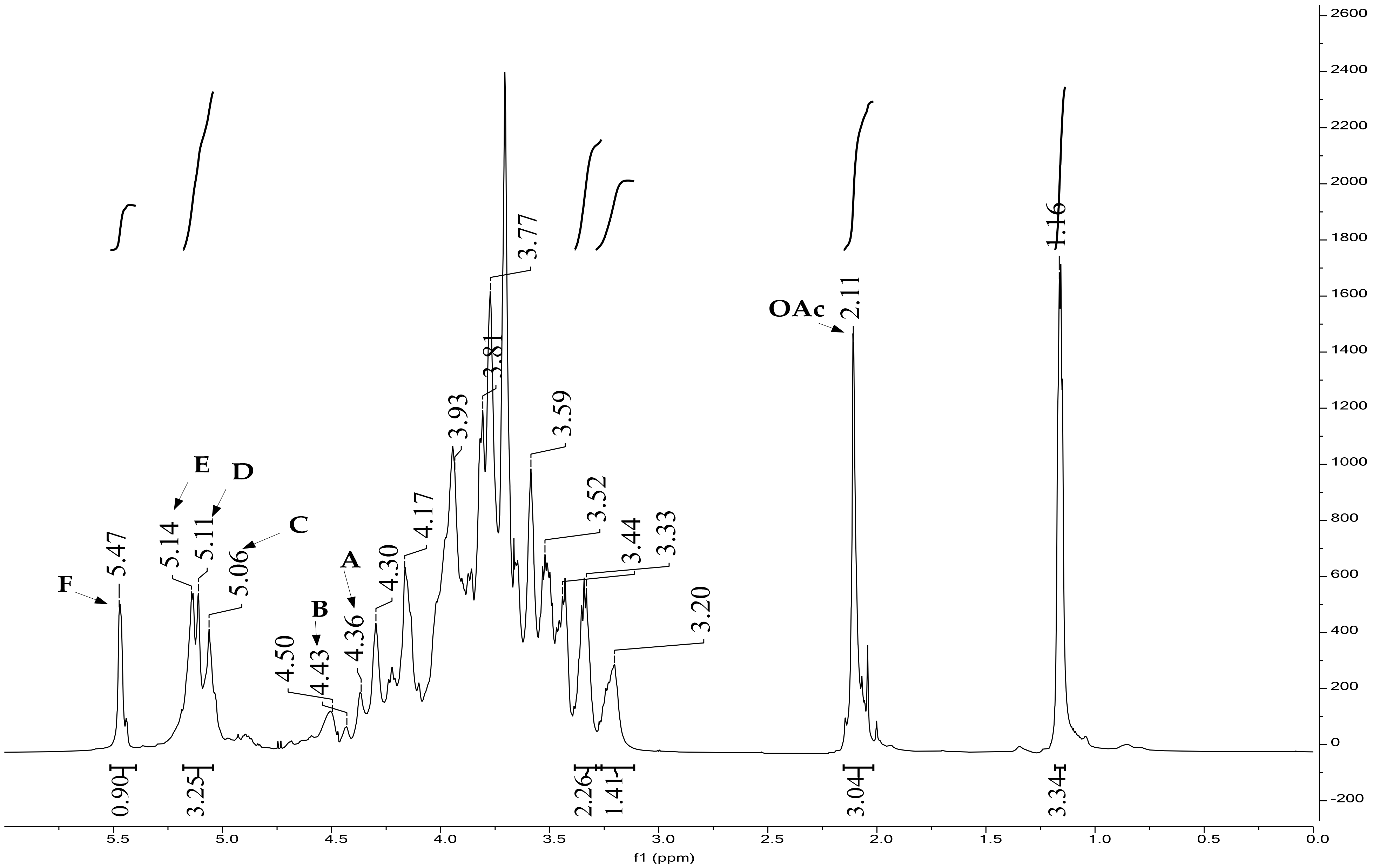

Figure S4. Full ^{1}H NMR spectrum (800 MHz) of TFP-F1 (D_2O, 323K).

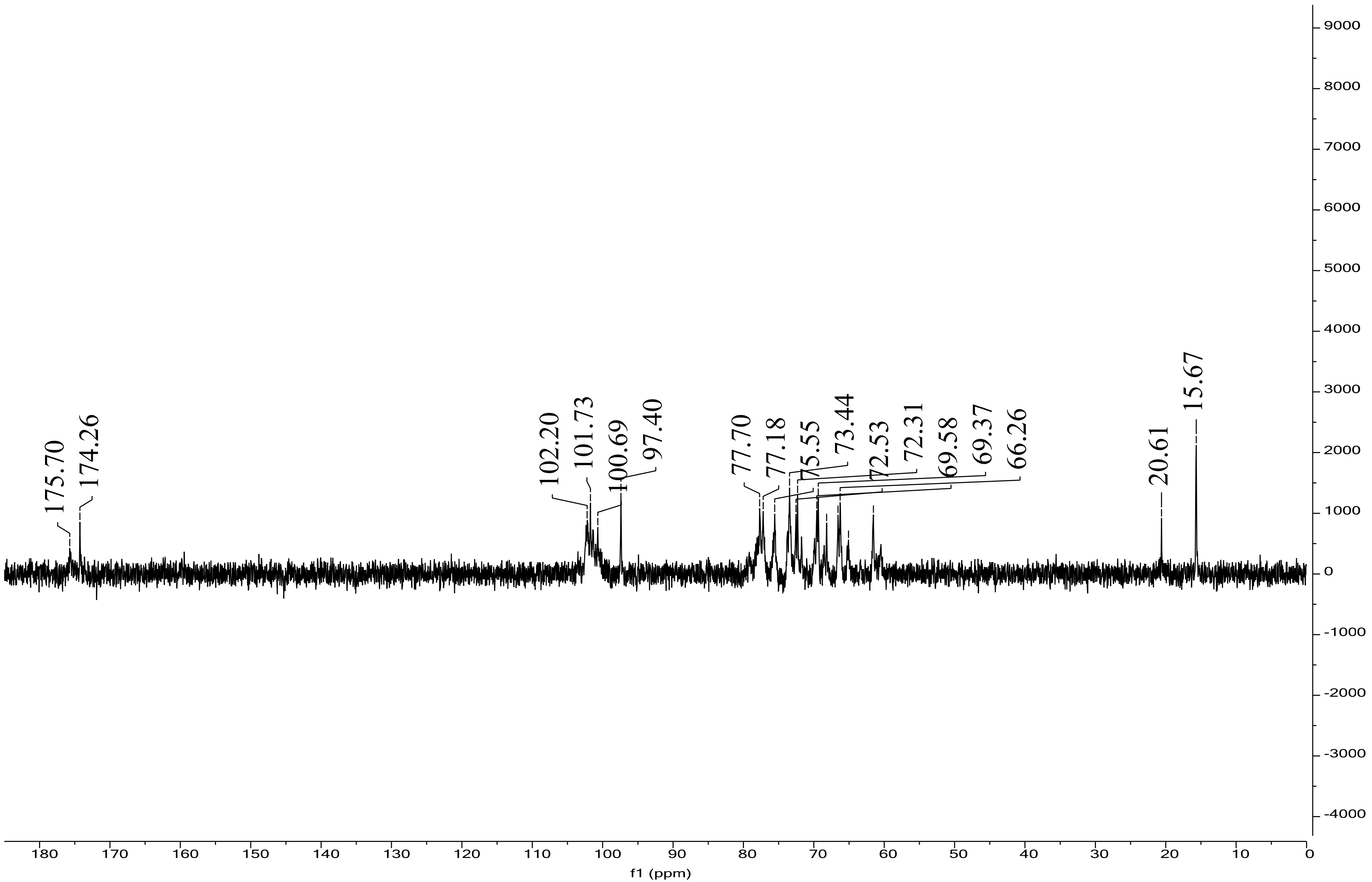

Fig. S5a. Full ^{13}C NMR spectrum (160 MHz) of TFP-F1 (D_2O, 323K).

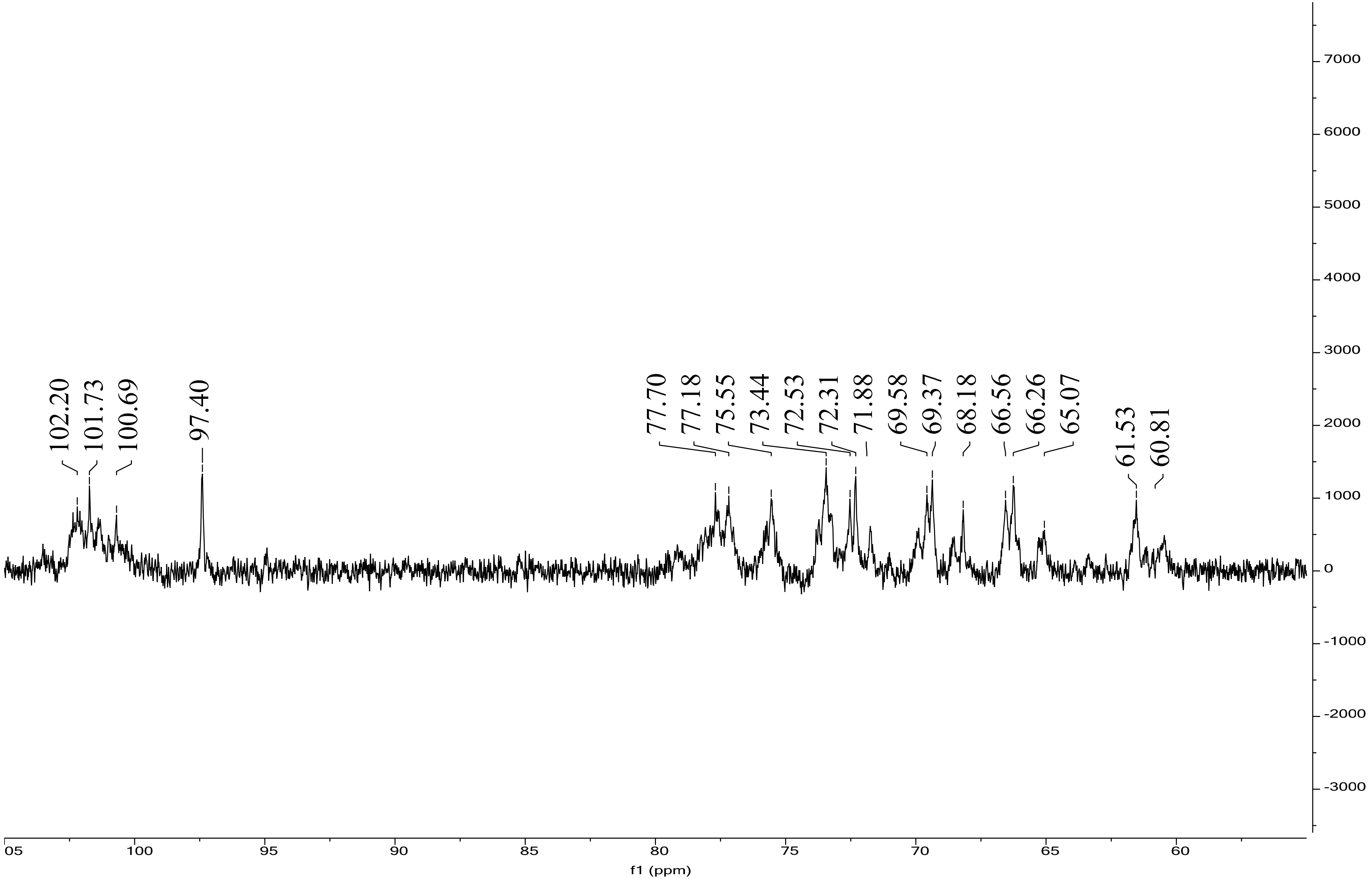

Figure S5b. Enlarged ^{13}C NMR spectrum (from 55 to 105 ppm) (160 MHz) of TFP-F1

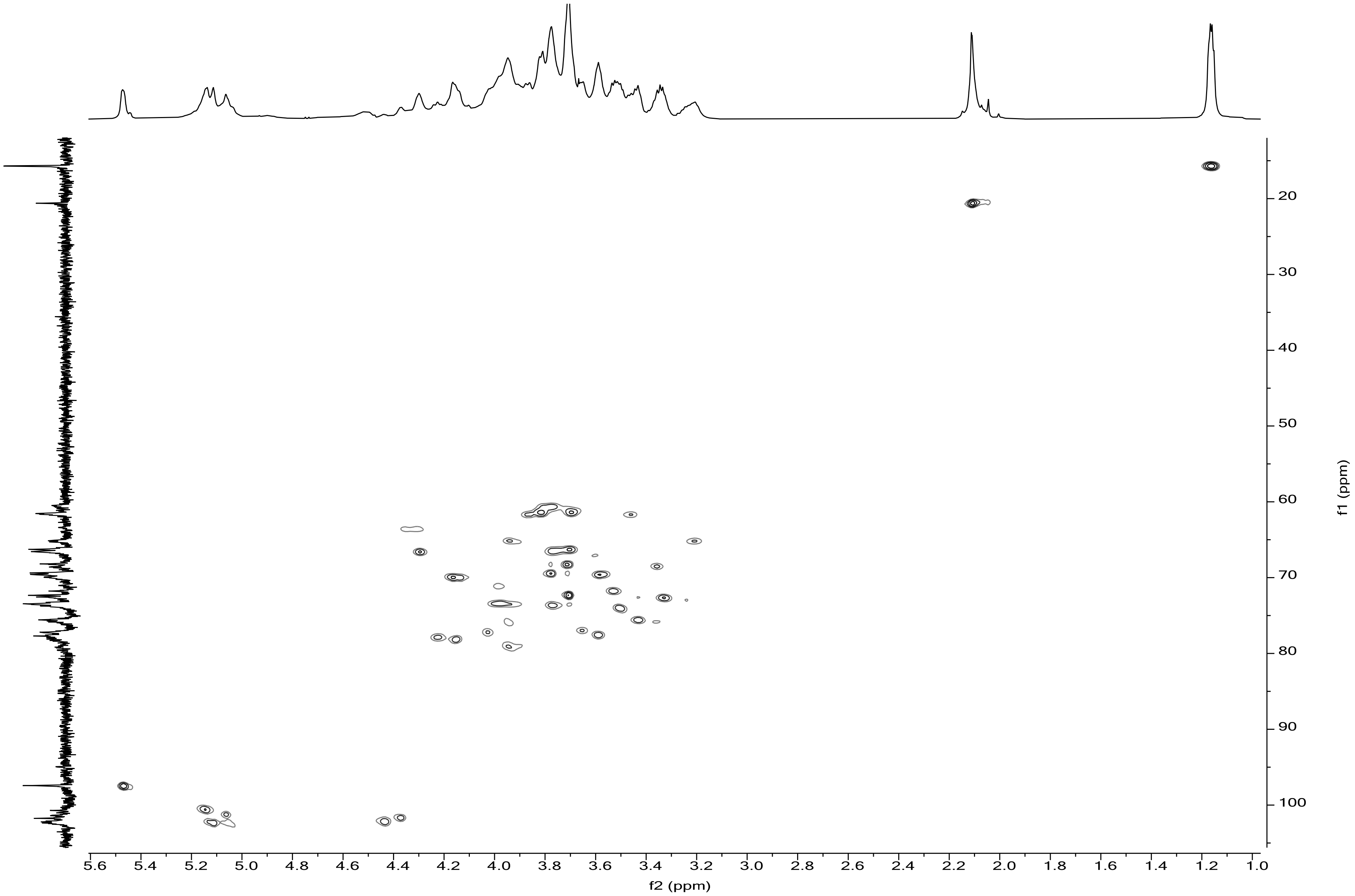

Figure S6a. Full HSQC spectrum of TFP-F1.

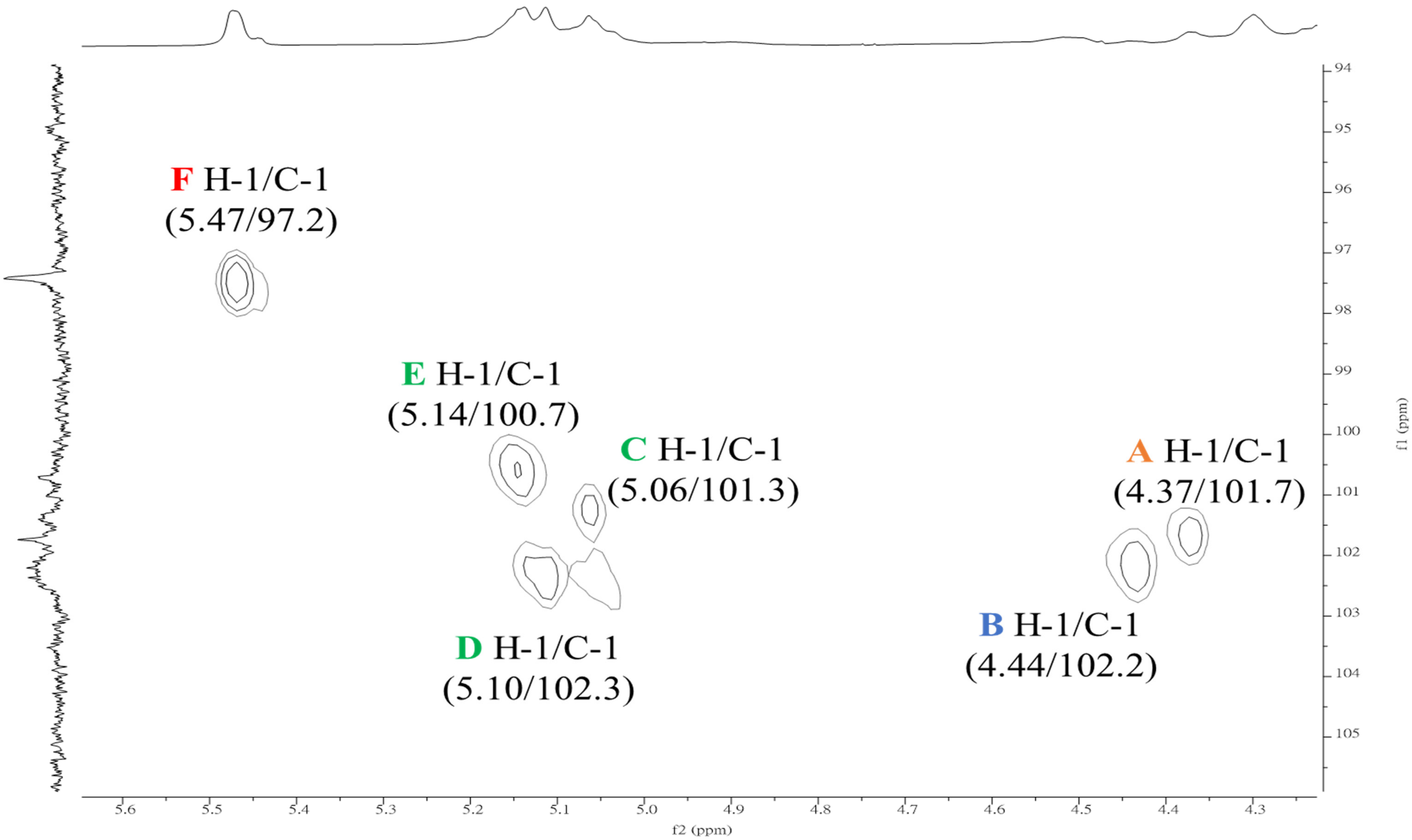

Figure S6b. Enlarged HSQC spectrum (I) of TFP-F1.

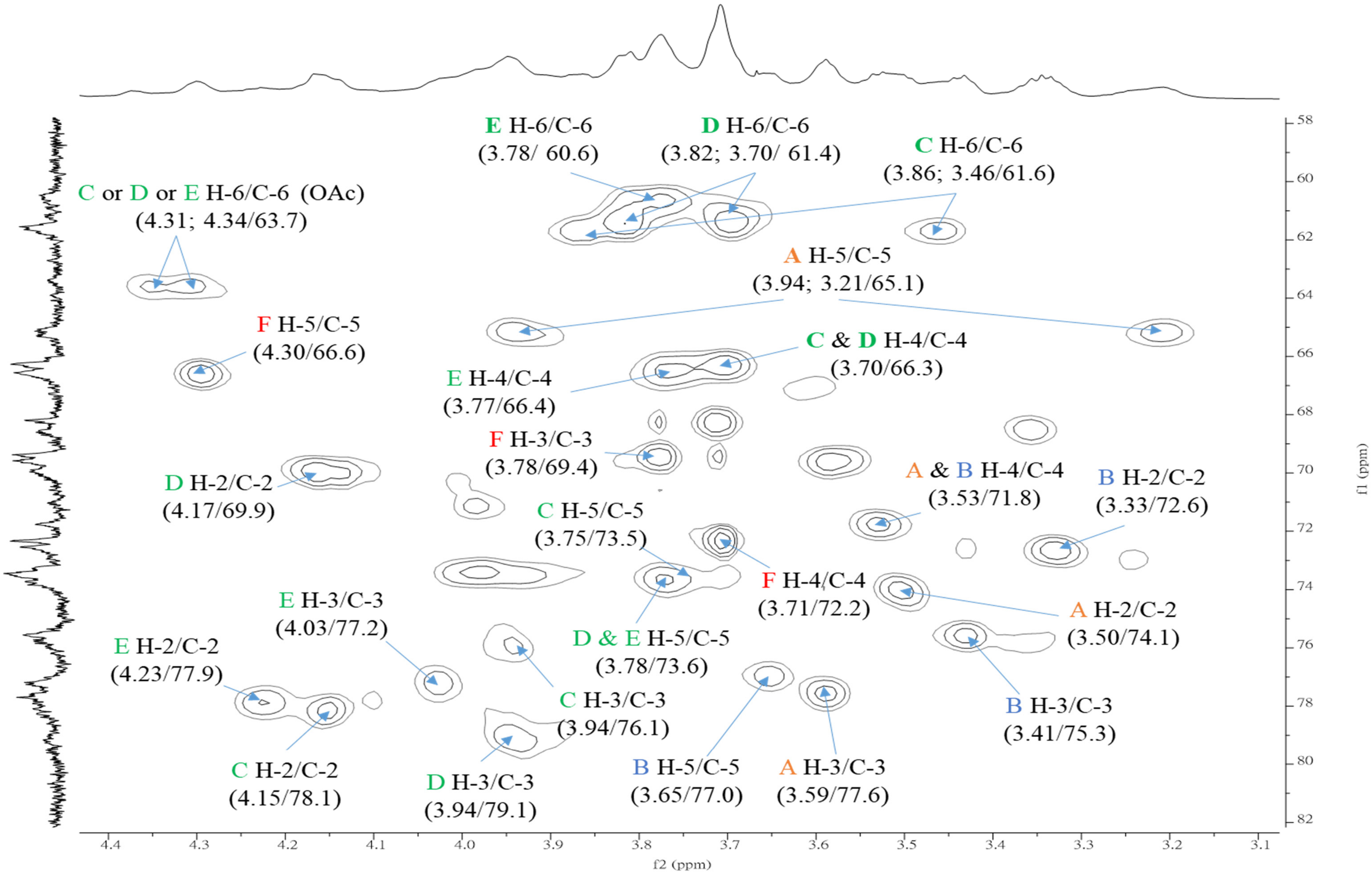

Figure S6c. Enlarged HSQC spectrum (II) of TFP-F1.

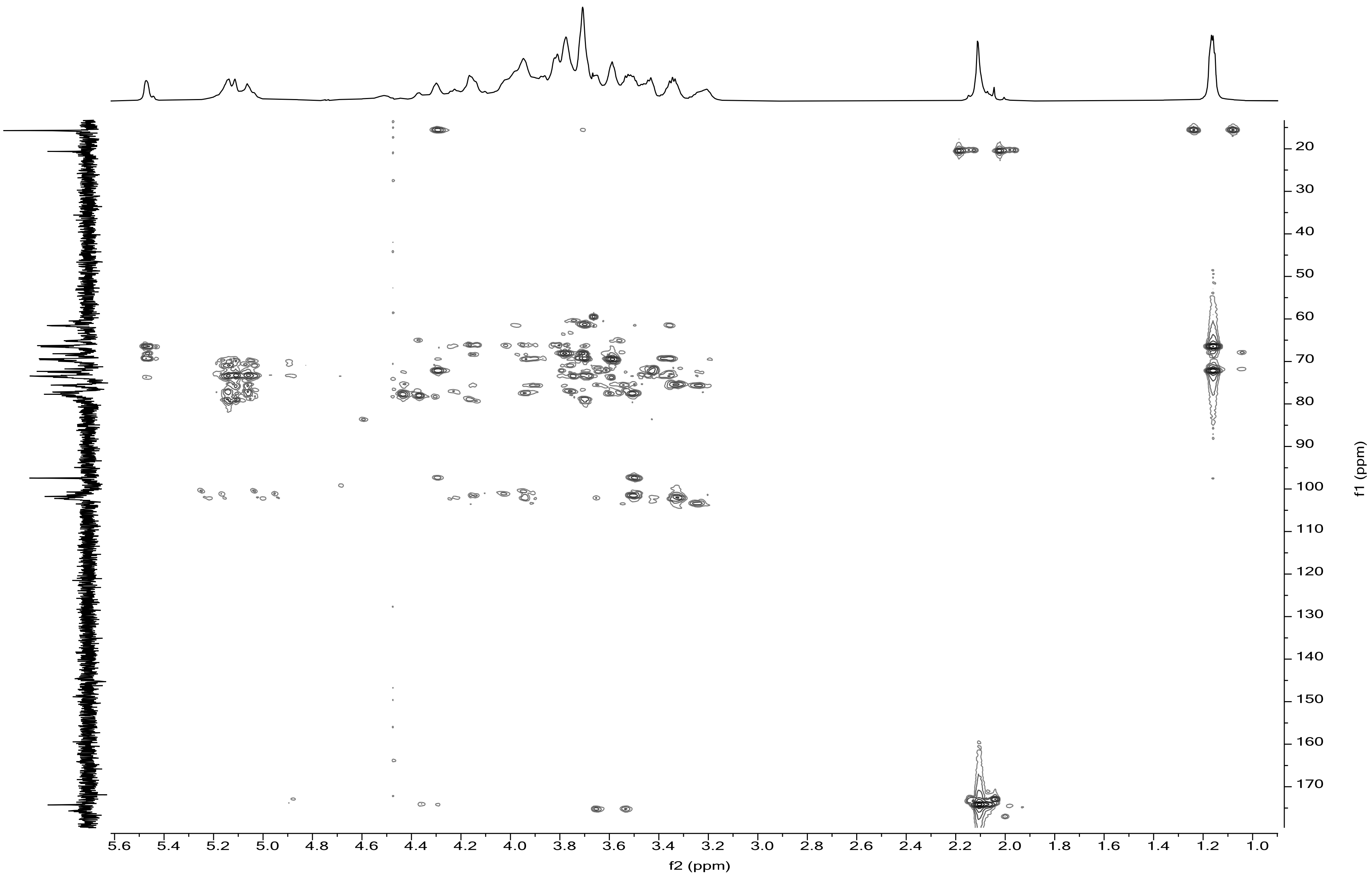

Figure S7a. Full HMBC spectrum of TFP-F1.

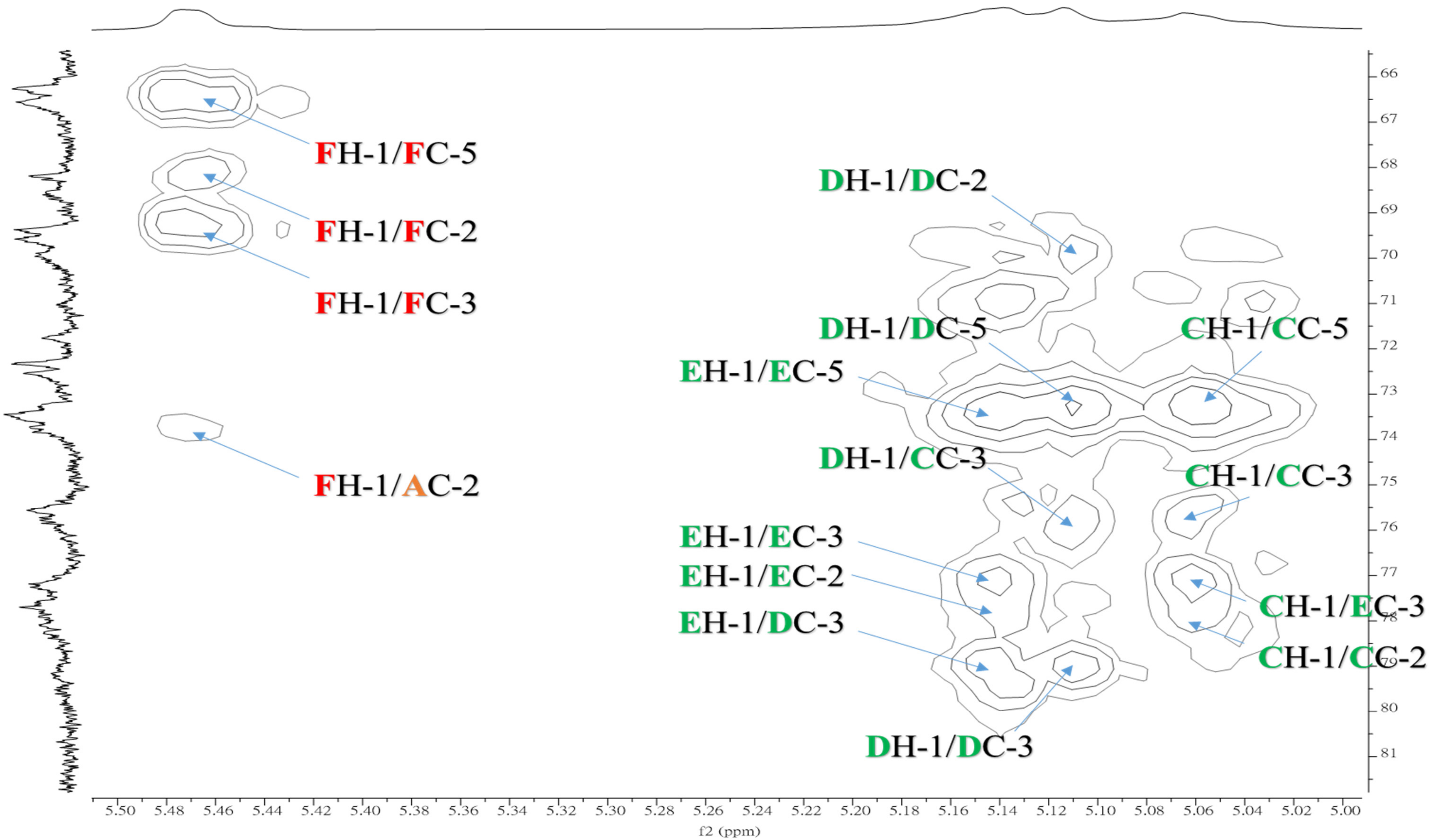

Figure S7b. Enlarged HMBC spectrum (I) of TFP-F1.

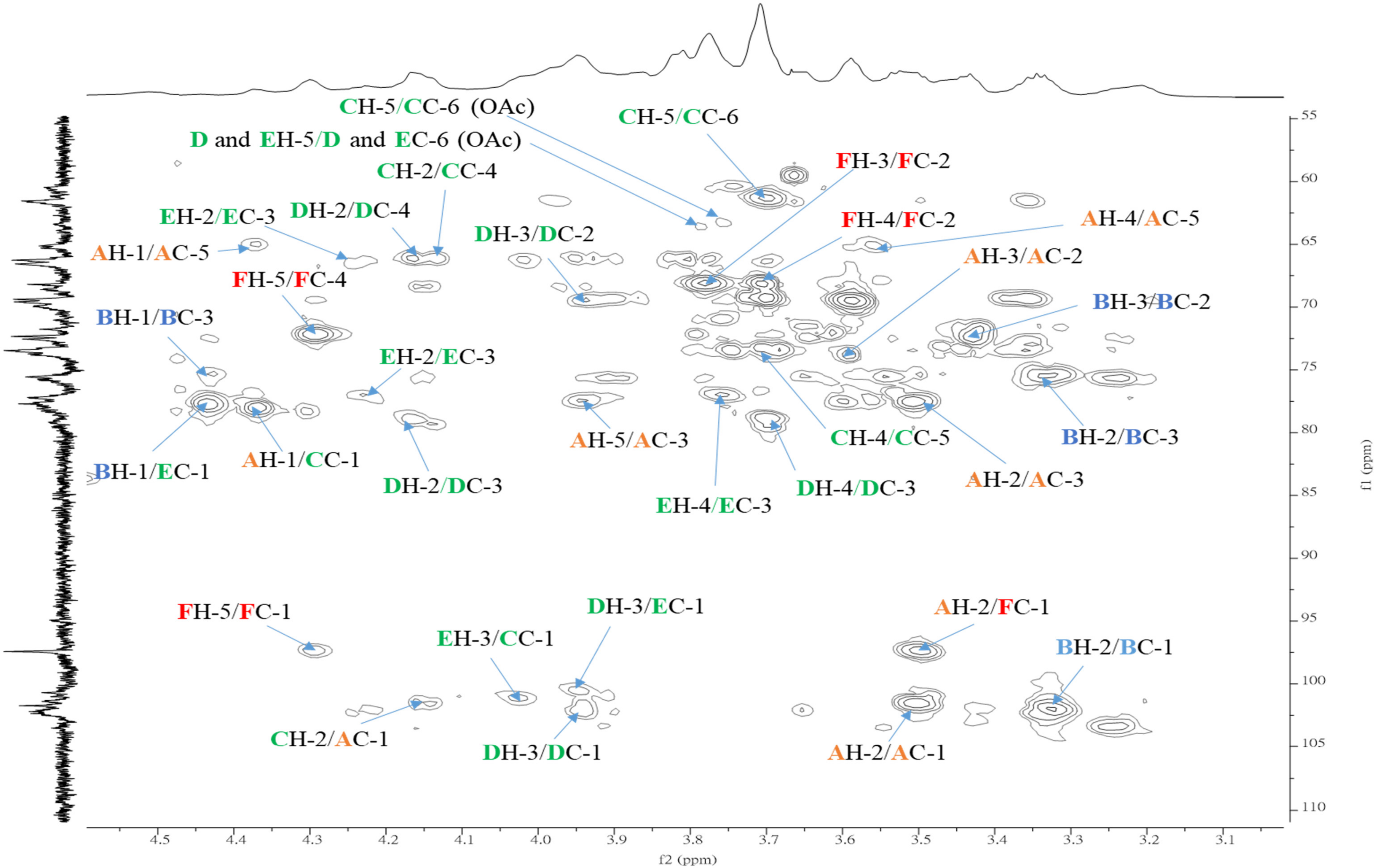

Figure S7c. Enlarged HMBC spectrum (II) of TFP-F1.

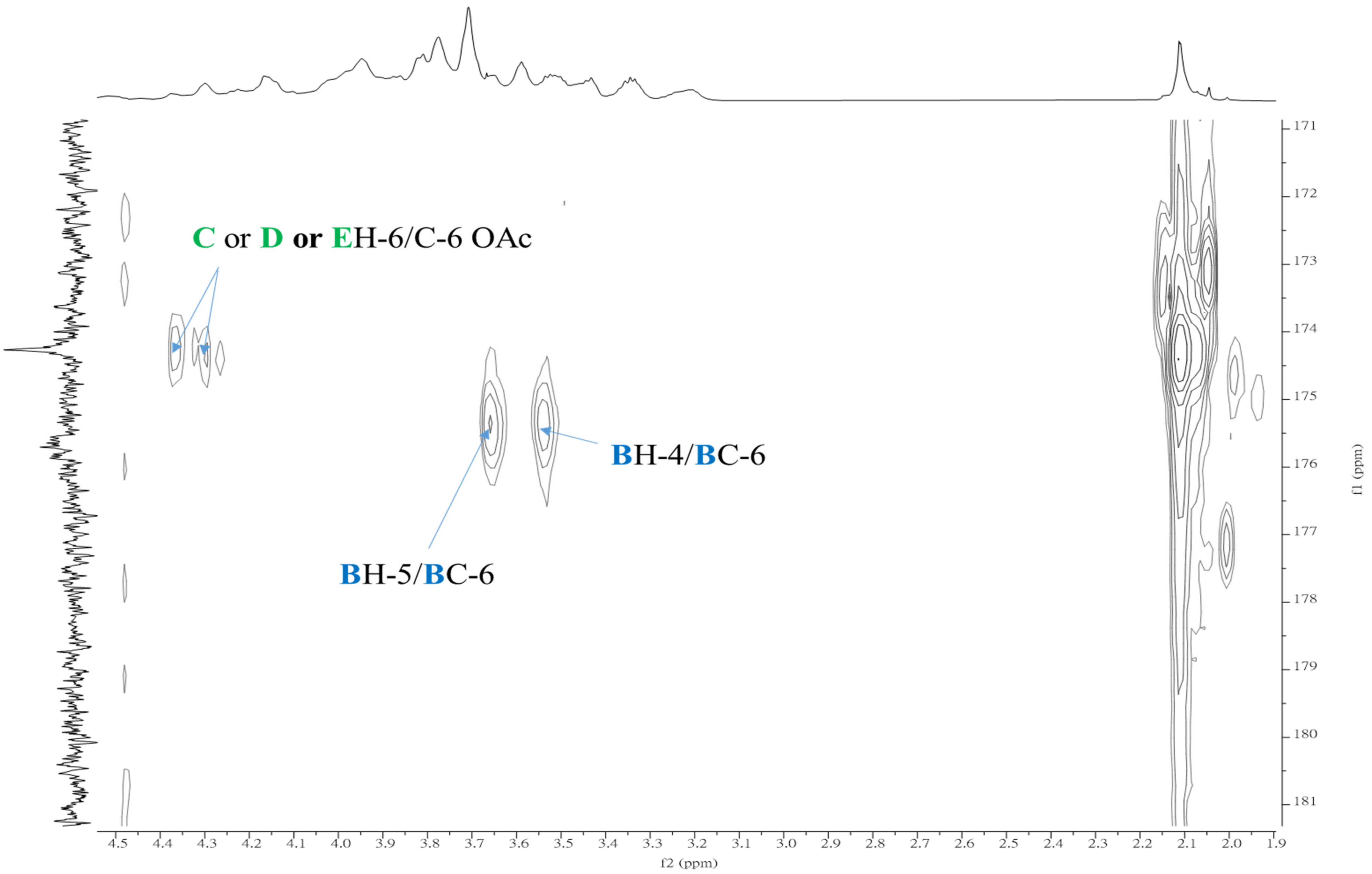

Figure S7d. Enlarged HMBC spectrum (III) of TFP-F1.

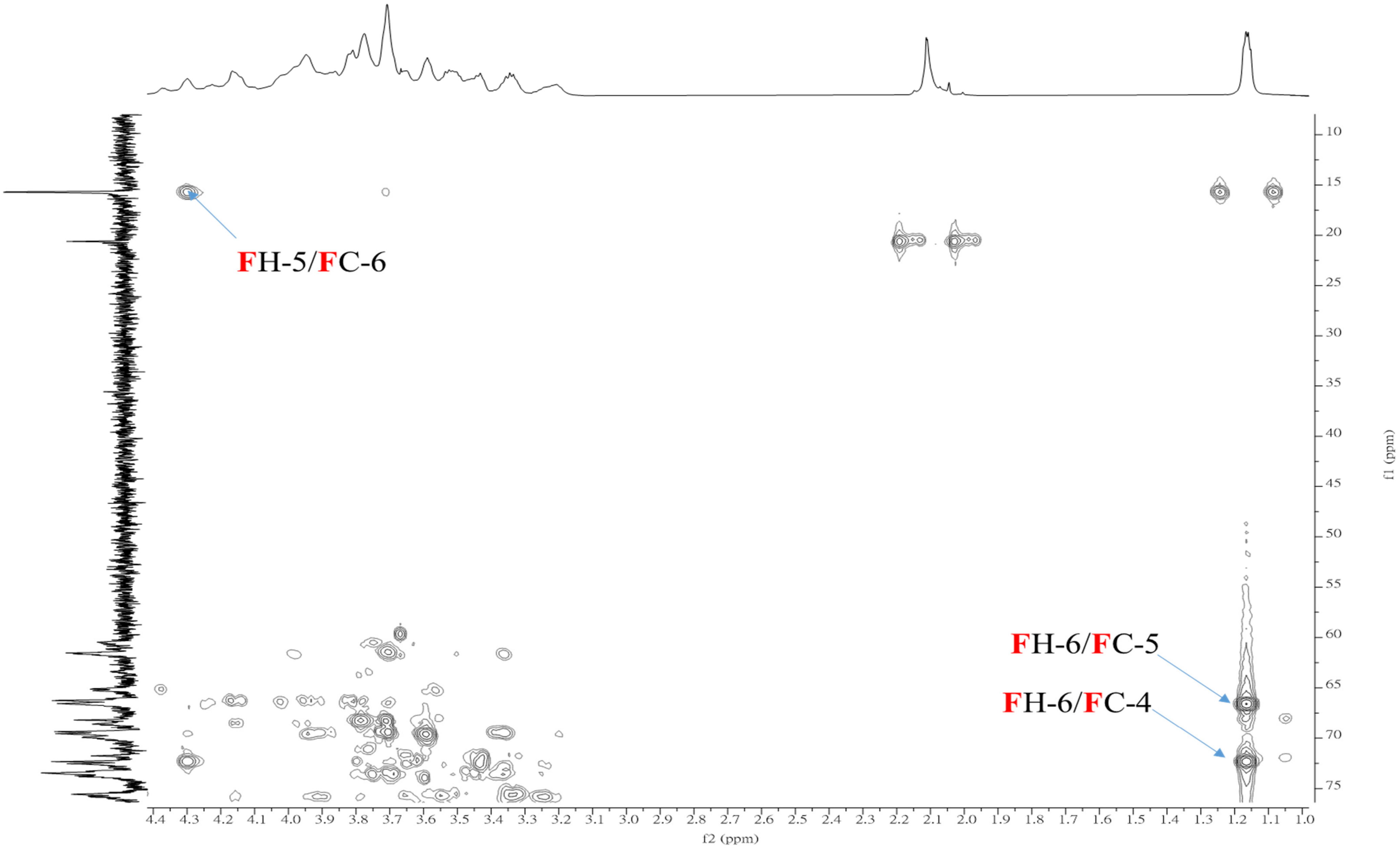

Figure S7e. Enlarged HMBC spectrum (IV) of TFP-F1.

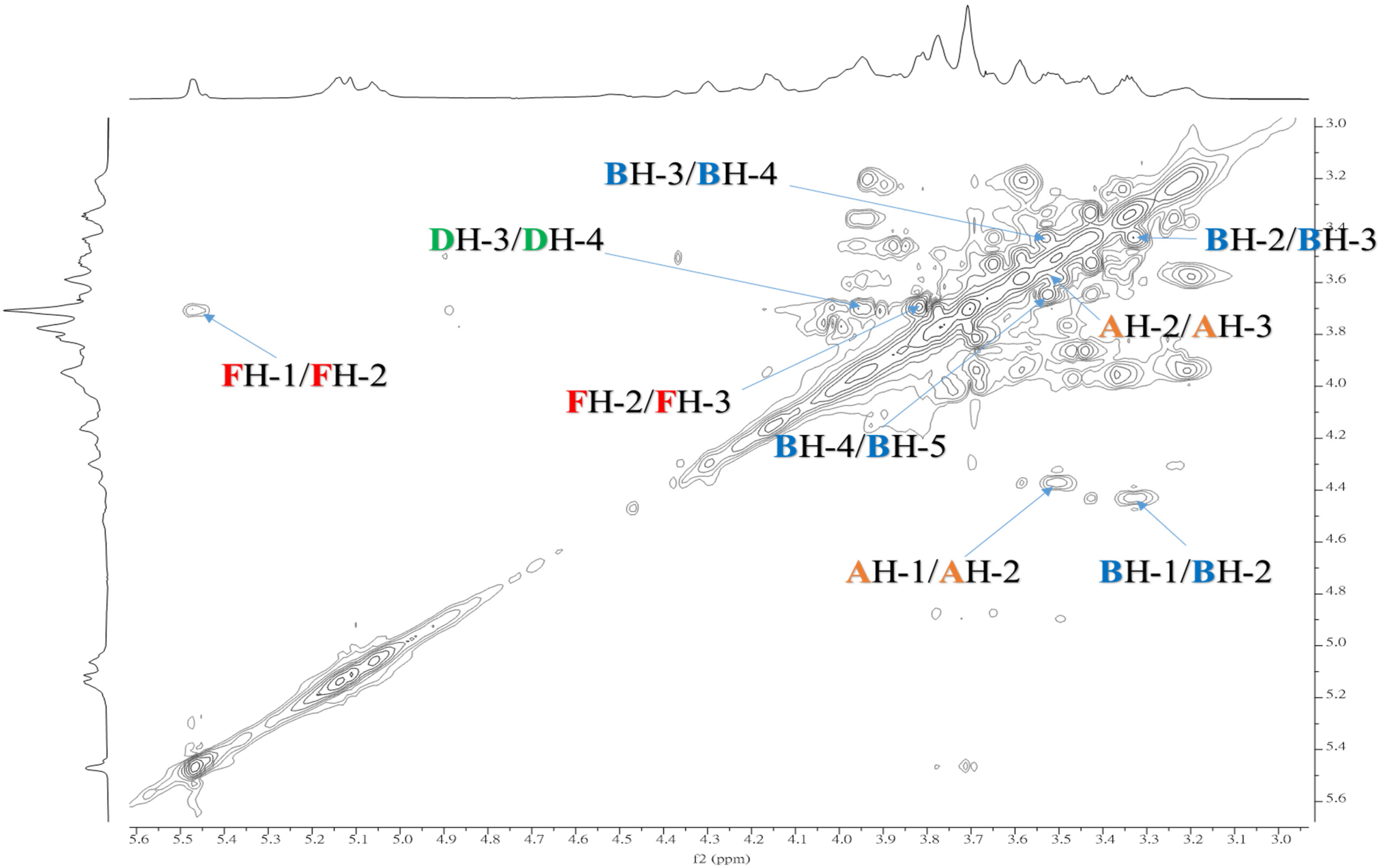

Figure S8. Full COSY spectrum of TFP-F1.

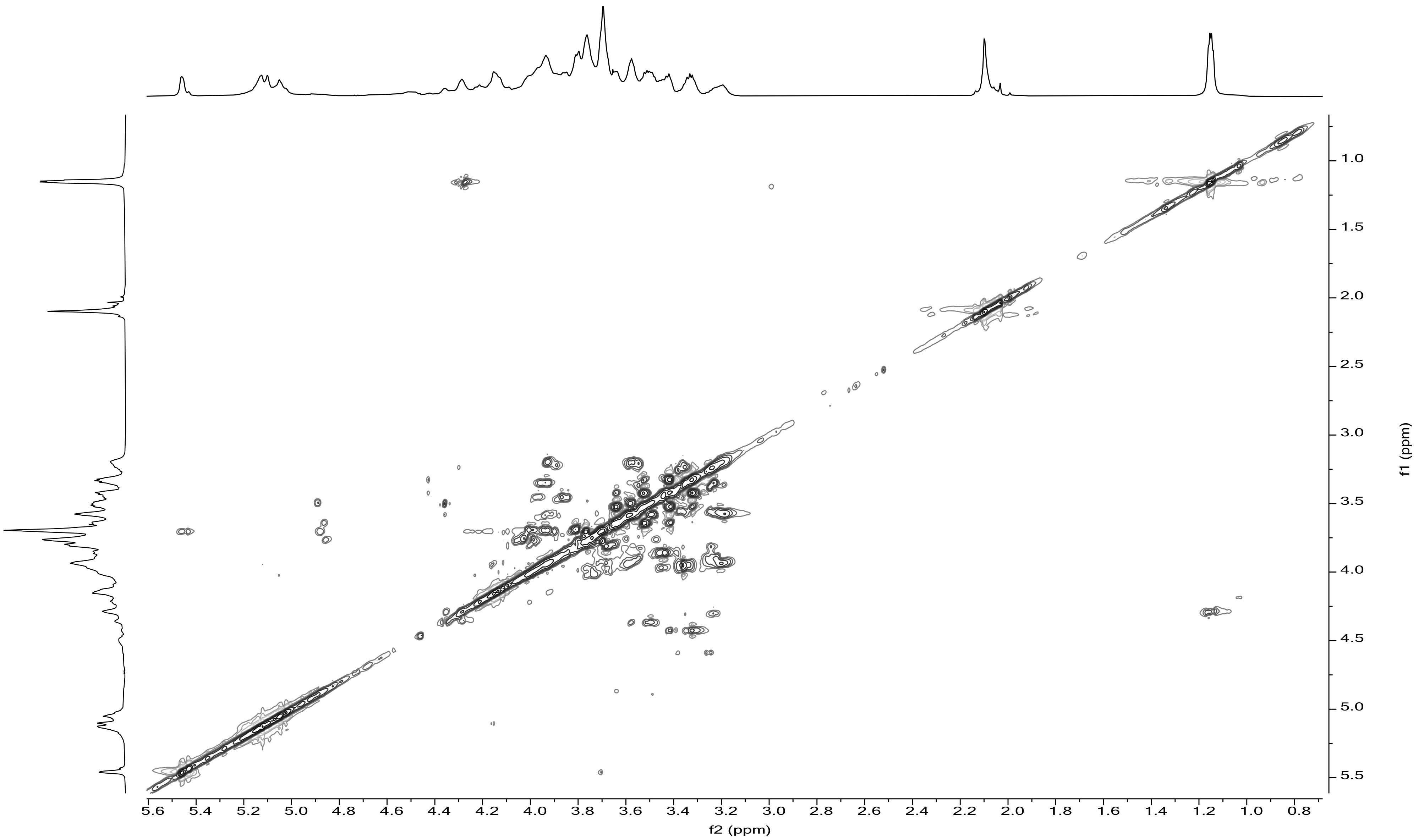

Figure S9a. Full TOCSY spectrum of TFP-F1.

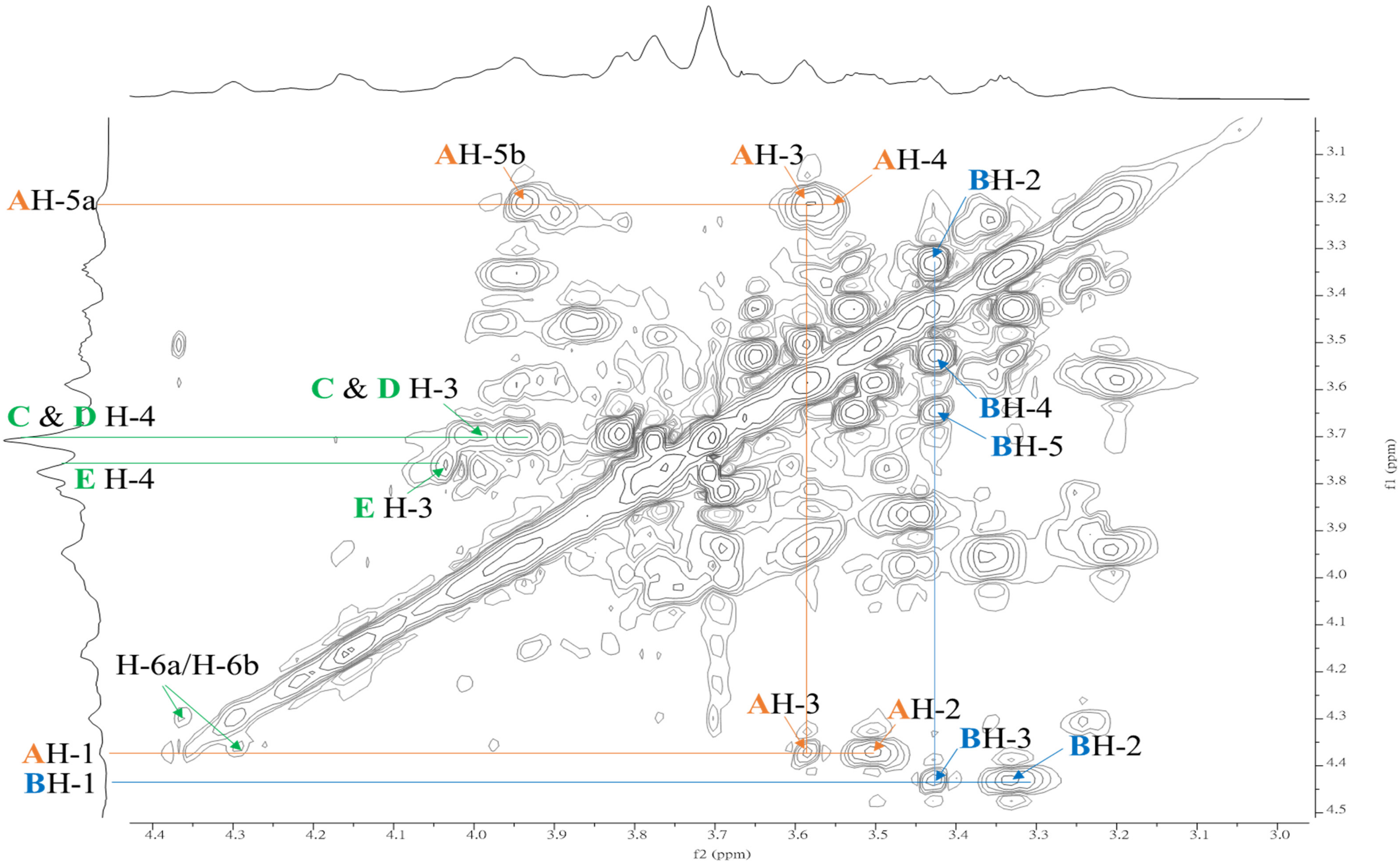

Figure S9b. Enlarged TOCSY spectrum of TFP-F1.

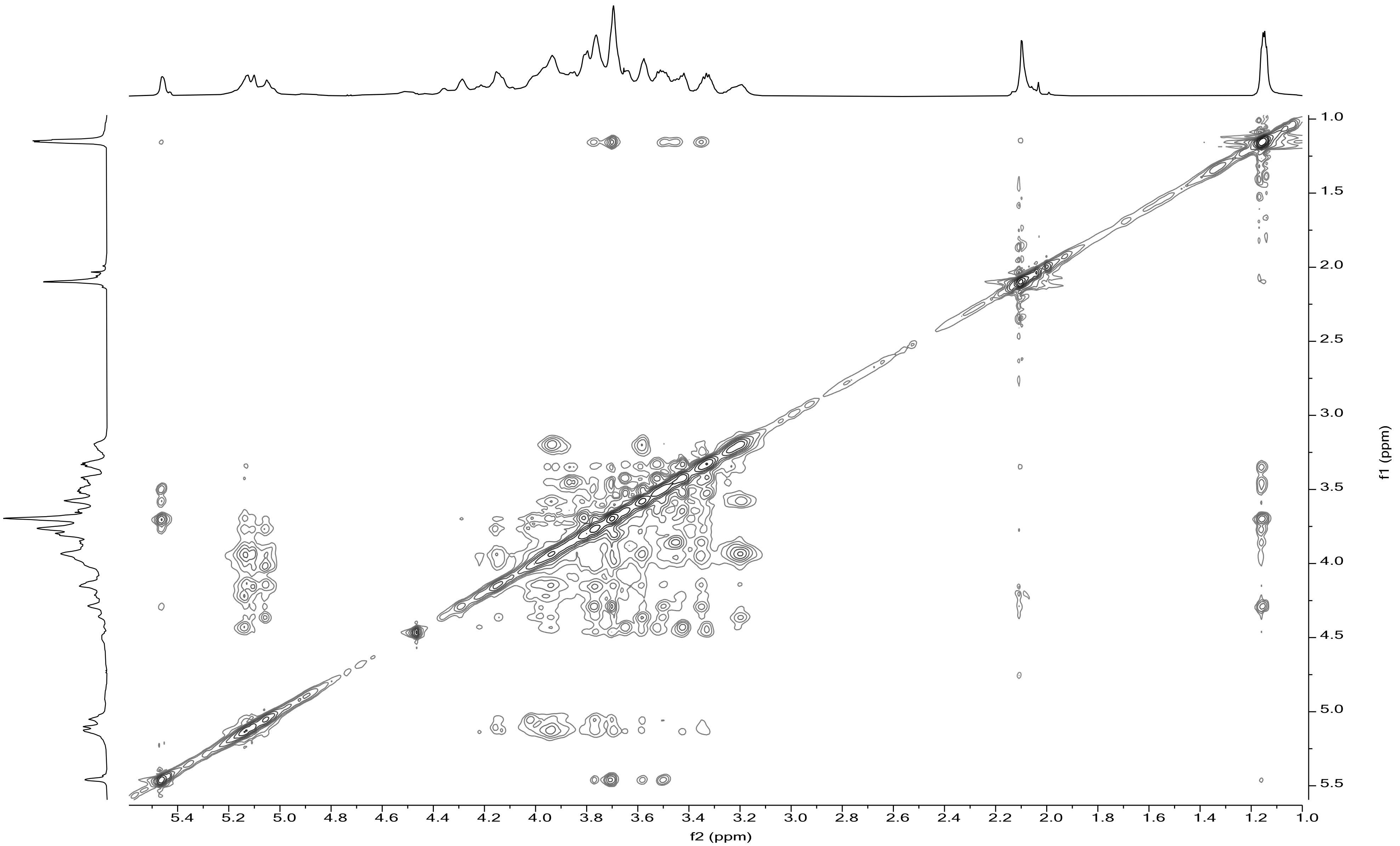

Figure S10a. Full NOESY spectrum of TFP-F1.

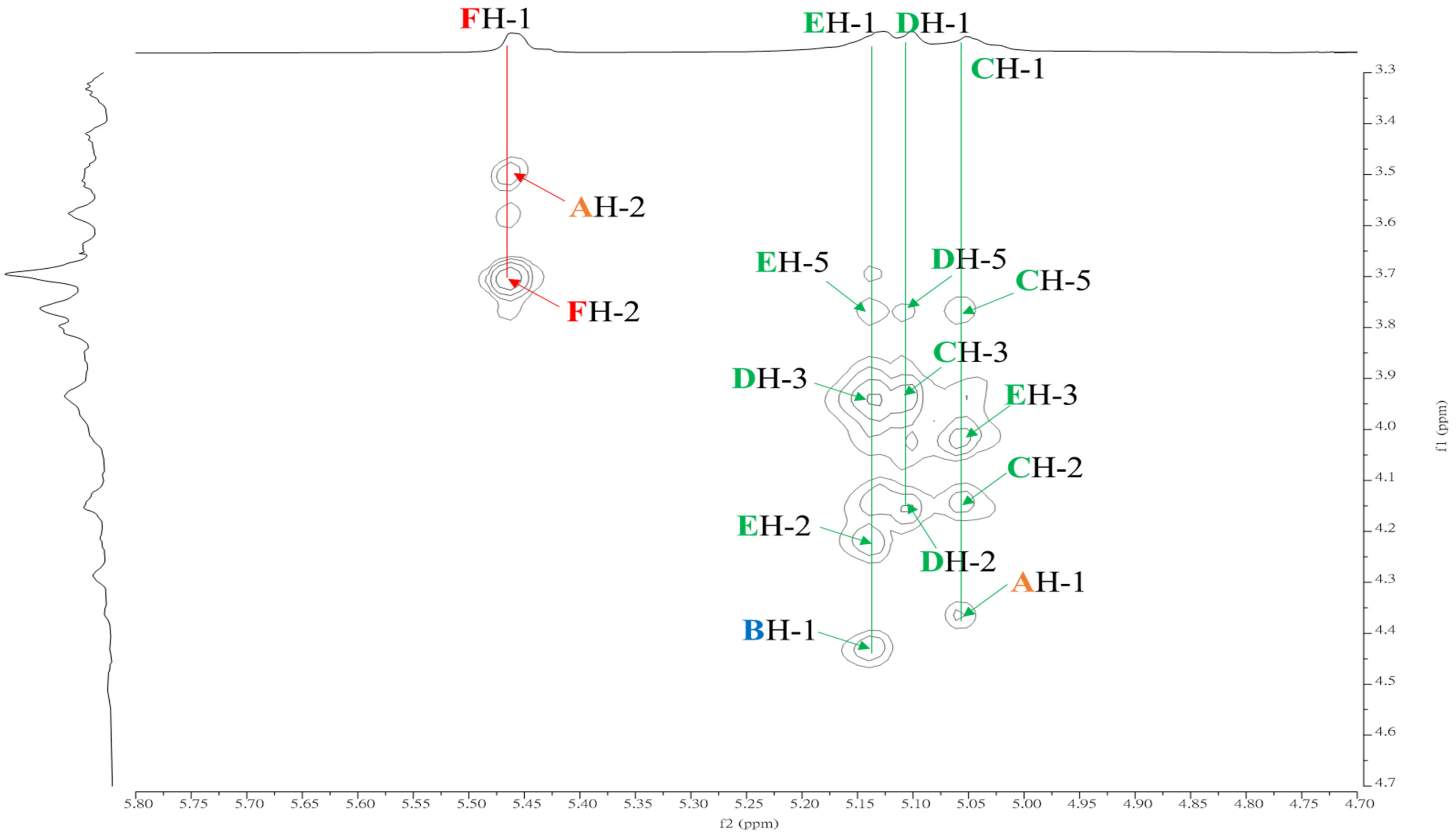

Figure S10b. Enlarged NOESY (I) spectrum of TFP-F1.

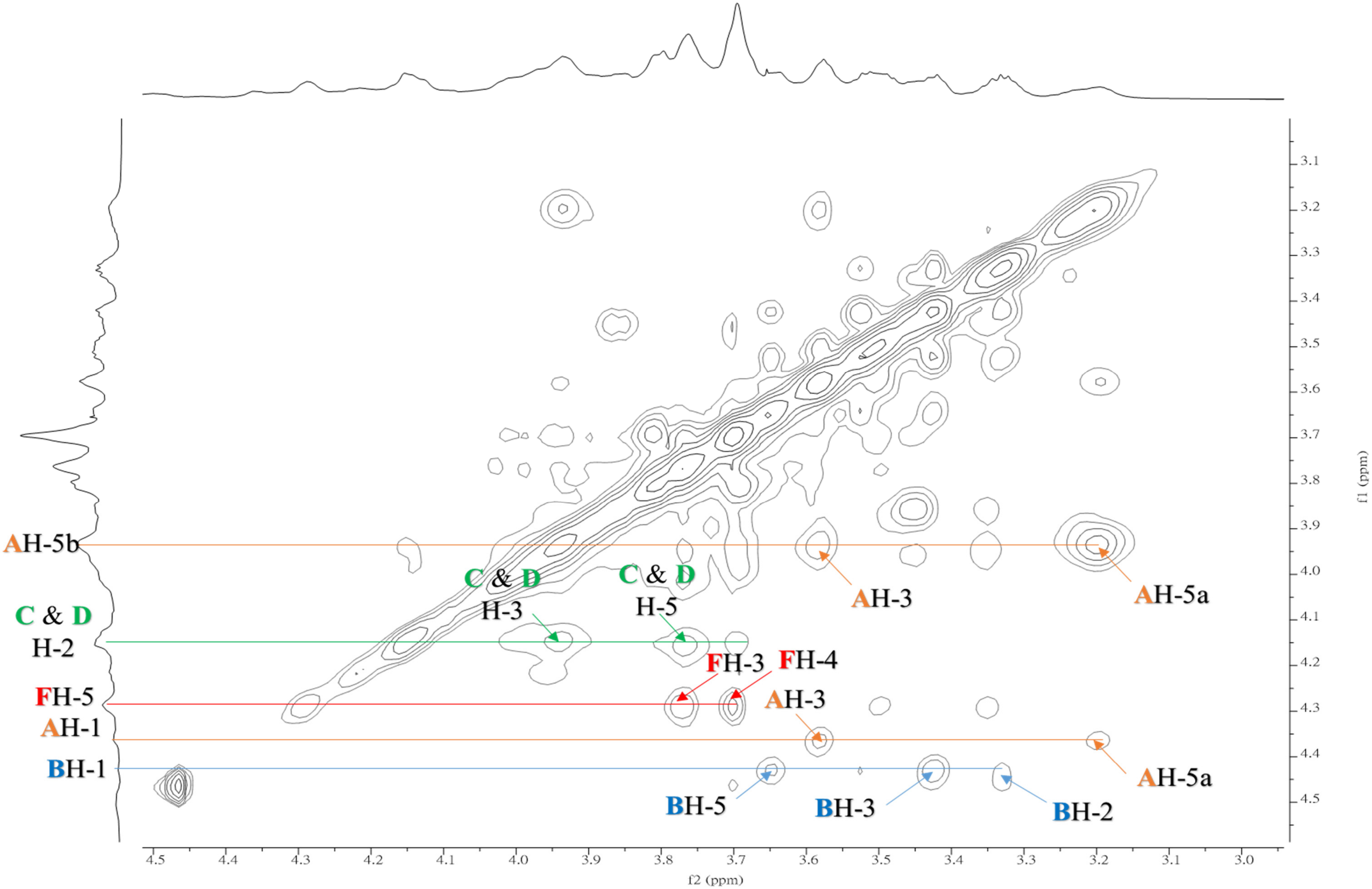

Figure S10c. Enlarged NOESY (II) spectrum of TFP-F1.

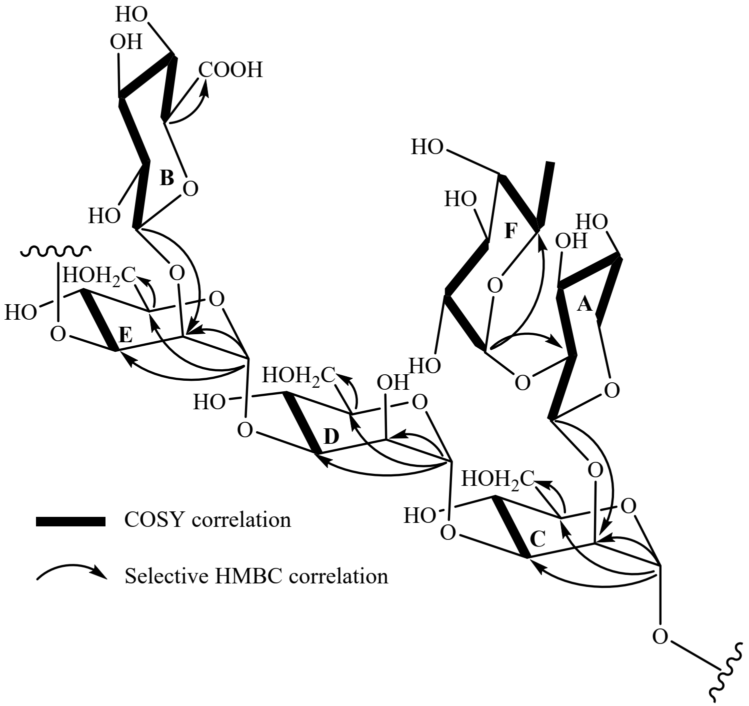

Figure S11. COSY and selective HMBC correlations of TFP-F1.

Table S4. Key NOE correlations of TFP-F1

Anomeric signals	NOE correlations (δ ppm)
A (4.37)	AH-3 (3.59); AH-5a (3.21); CH-1 (5.06)
B (4.44)	BH-2 (3.33); BH-3 (3.41); BH-5 (3.65); EH-1 (5.13)
C (5.06)	CH-2 (4.15); CH-3 (3.94); CH-5 (3.75) AH-1(4.37); EH-3 (4.03)
D (5.10)	DH-2 (4.17); DH-5 (3.78); CH-3 (3.94)
E (5.13)	EH-2 (4.23); EH-5 (3.78); DH-3 (3.94); BH-1 (4.44)
F (5.47)	FH-2 (3.72); AH-2 (3.50)

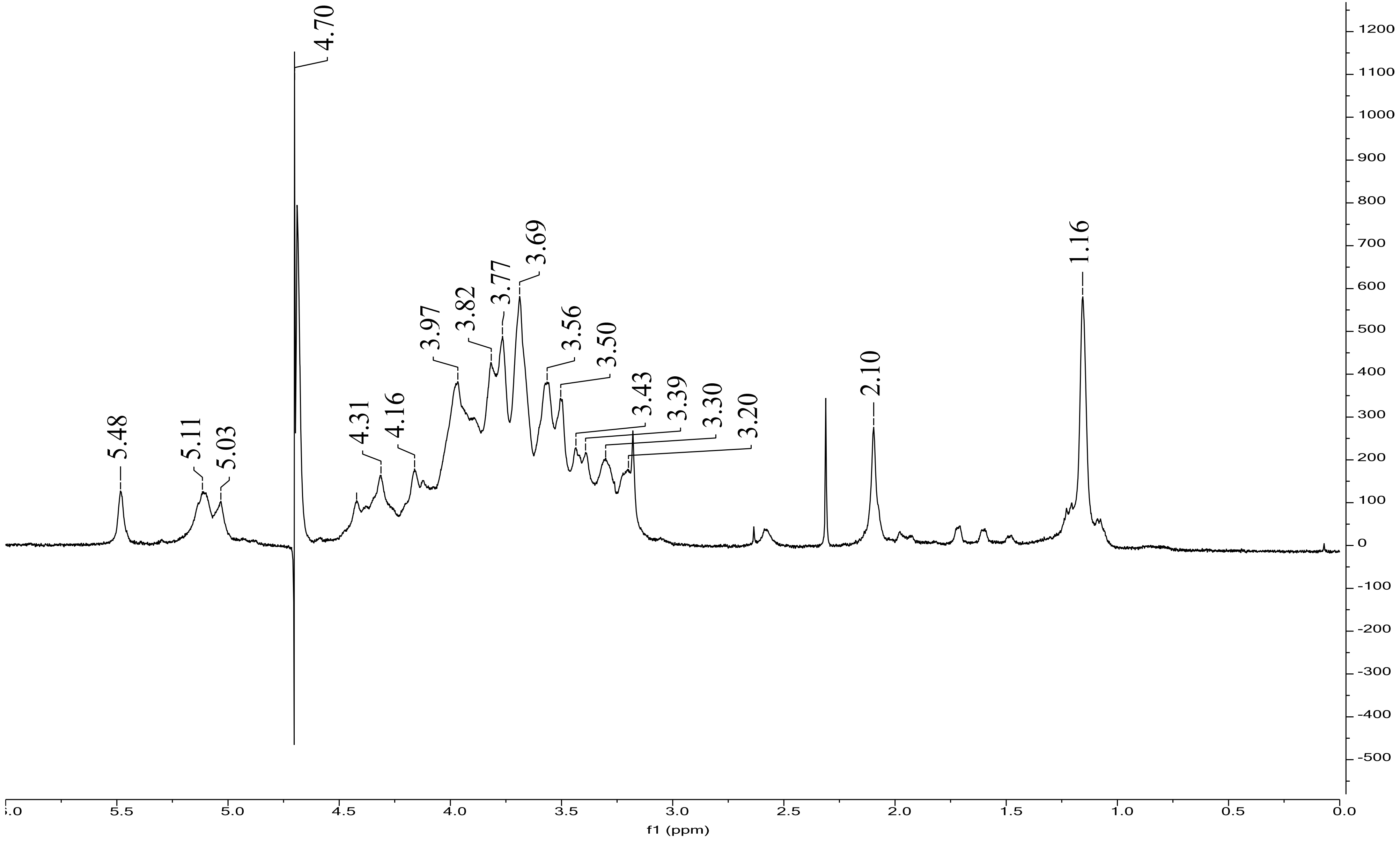

Figure S12. Full ^{1}H NMR spectrum (800 MHz) of TFP-F1R (D_2O, 298K)

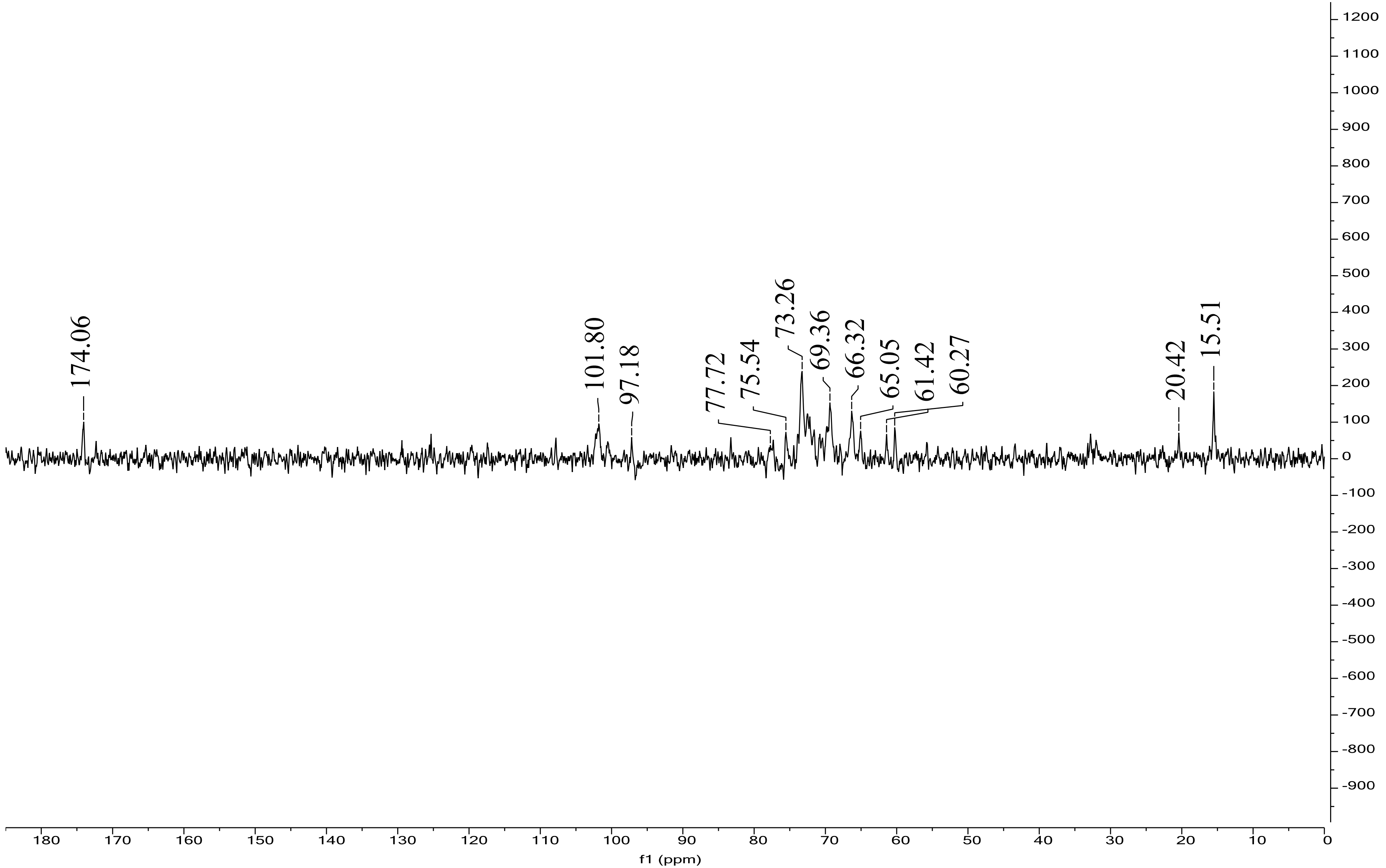

Figure S13. Full ^{13}C NMR spectrum (160 MHz) of TFP-F1R (D_2O, 298K)

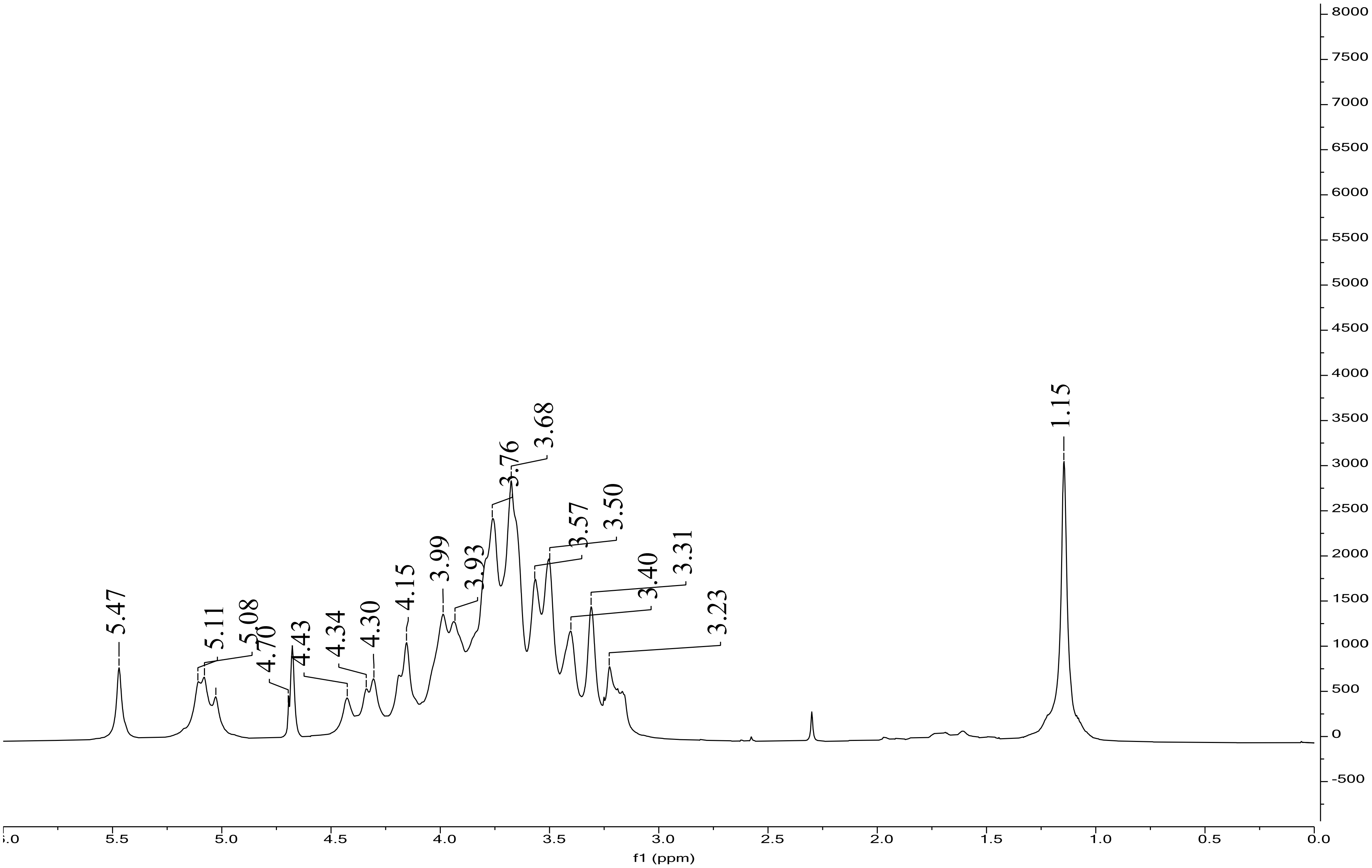

Figure S14. Full ^{1}H NMR spectrum (800 MHz) of deOAc TFP-F1R (D_2O, 298K)

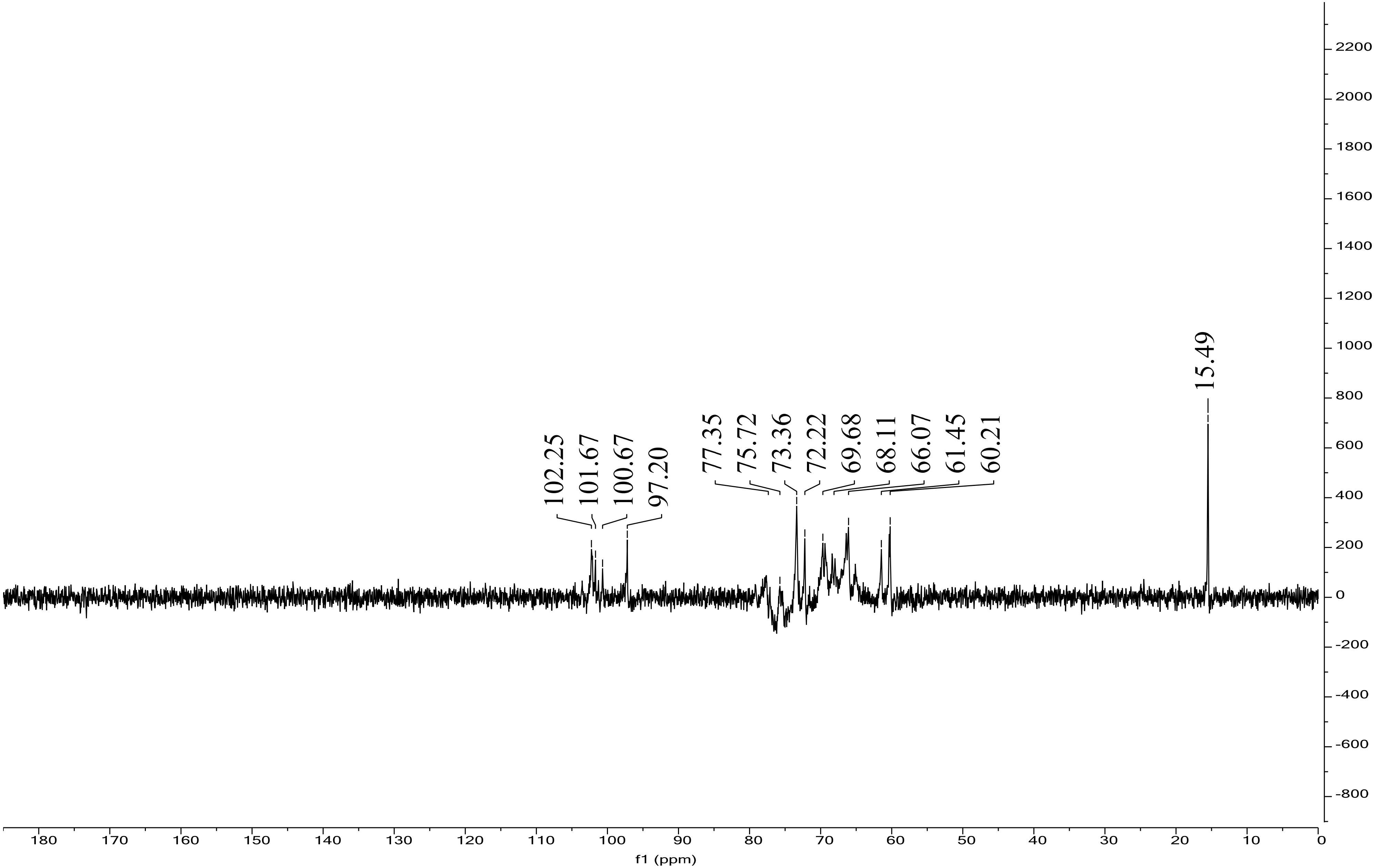

Figure S15. Full ^{13}C NMR spectrum (160 MHz) of deOAc TFP-F1R (D_2O, 298K)

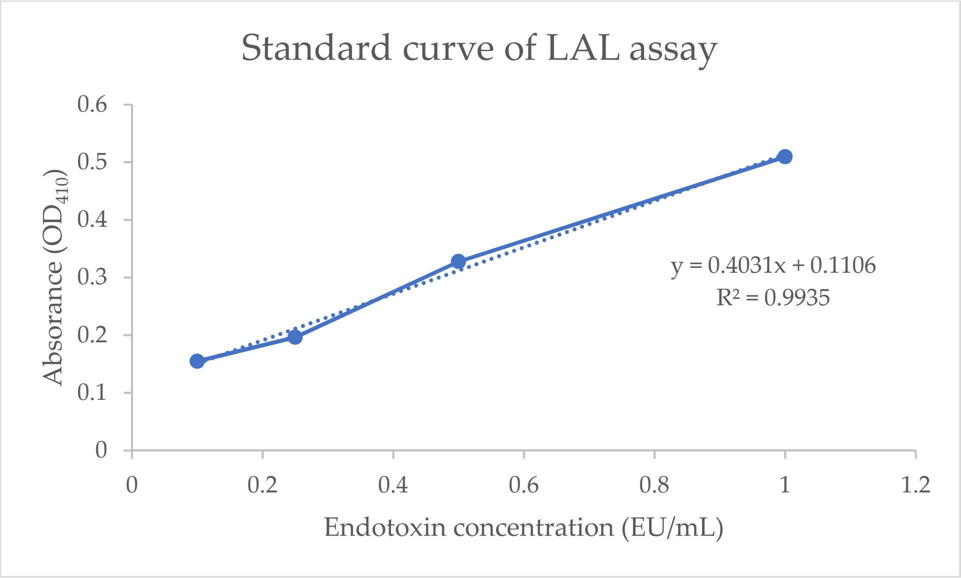

Figure S16. The standard curve of LAL assay. The *E. coli* endotoxin was prepared for standards of different concentrations (0.1, 0.25, 0.5, and 1.0 EU/mL).

Table S5. The endotoxin quantity of TFP-F1 and its derivatives

Sample	Endotoxin concentration (EU/mL)	Standard deviation
TFP-F1 (1 μg/mL)	0.079053998	0.015889224
TFP-F1R (1 μg/mL)	0.177540712	0.031779415
deOAc TFP-F1R (1 μg/mL)	-0.004051926	0.007885321

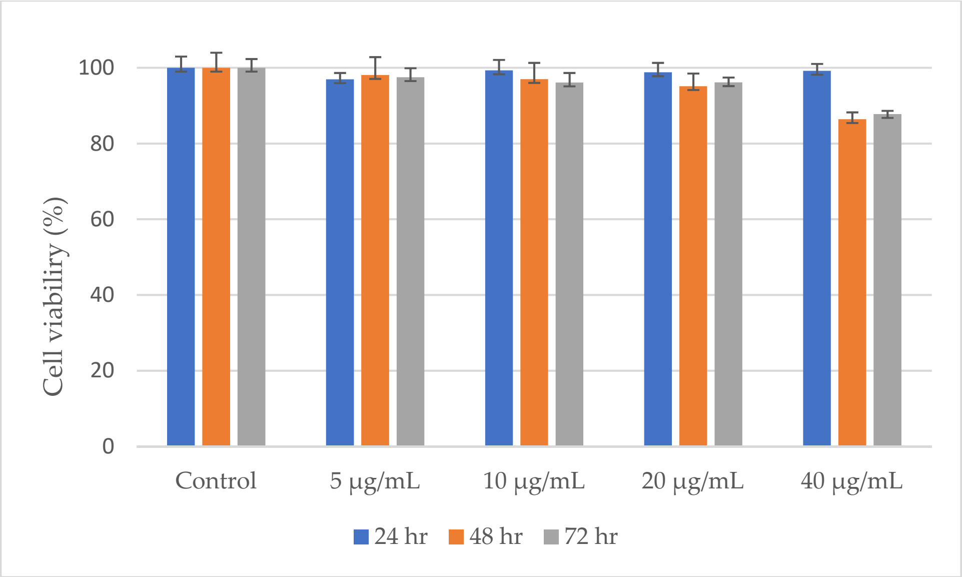

Figure S17. Cell viability was measured by Alamar blue assay. J774A.1 cells were treated with different concentrations (5, 10, 20, and 40 μg/mL) of TFP-F1 for 24 h, 48 h, and 72 h.
